# coreSCD: multi-stakeholder consensus on core outcomes for sickle cell disease clinical trials

**DOI:** 10.1186/s12874-021-01413-8

**Published:** 2021-10-19

**Authors:** Ellen Tambor, Matoya Robinson, Lewis Hsu, Hsing-Yuan Chang, Jennifer Al Naber, on behalf of the coreSCD Panel

**Affiliations:** 1 Center for Medical Technology Policy, 401 E. Pratt St., Suite 631, Baltimore, MD 21202 USA; 2 Micromattie Consulting, San Diego, CA USA; 3 Department of Pediatrics, University of Illinois at Chicago, Chicago, IL USA

**Keywords:** Sickle cell disease, Outcomes, Clinical trials, Stakeholder engagement

## Abstract

**Background:**

With the dramatic increase in the pipeline for new sickle cell disease (SCD) therapies in recent years, the time is ripe to ensure a robust body of evidence is available for decision making by regulators, payers, clinicians, and patients. Harmonization of the outcomes selected across interventional trials enables optimal post-trial appraisal and decision making through valid pooled analyses and indirect comparisons. We employed a structured, multi-stakeholder consensus process to develop core outcome sets (COS) for use in clinical trials of SCD interventions.

**Methods:**

CoreSCD utilized a modified Delphi method adapted from the standards recommended by the Core Outcome Measures in Effectiveness Trials (COMET) Initiative. An initial list of candidate outcomes was developed through a targeted literature review and input from an 11-member advisory committee. A 44-member multi-stakeholder Delphi Panel was established and included patients and family members, advocates, clinicians, researchers, payers, health technology assessors, representatives from government agencies, and industry representatives. Patients/advocates comprised 25% of the Delphi Panel and orientation and training was provided prior to the consensus process to ensure all were prepared to participate meaningfully. Panelists completed three rounds of an online survey to rate the importance of candidate outcomes for inclusion in the COS. Summary data was provided between each voting round and an in-person consensus meeting was held between the second and third round of voting. Consensus rules were applied following each round of voting to eliminate outcomes that did not meet predetermined criteria for retention.

**Results:**

Consensus was reached for two core outcome sets. The final COS for trials of disease-modifying therapies includes ten outcomes and the COS for trials of acute interventions includes six outcomes. Both core sets include clinical outcomes as well as outcomes related to functioning/quality of life, resource utilization, and survival/mortality.

**Conclusions:**

Use of the COS in clinical development programs for SCD will help to ensure that relevant, consistent outcomes are available for decision making across the product lifecycle.

**Supplementary Information:**

The online version contains supplementary material available at 10.1186/s12874-021-01413-8.

## Background

Sickle cell disease (SCD) is a group of inherited red blood cell disorders that affect approximately 100,000 individuals in the United States (US) [1]. Worldwide, an estimated 300,000 babies are born with SCD each year [2]. In the US and other high-income countries, newborn screening and intervention have resulted in a significant increase in life expectancy [2]. However, people living with SCD face a wide range of acute and chronic complications including acute pain episodes, increased susceptibility to infections, stroke, anemia, organ damage, and chronic pain [3, 4]. Although disease severity varies widely, SCD can have a significant impact on quality of life and managing the disease over a lifetime is associated with high health care utilization and cost [5, 6].

Until recently, treatment options for individuals living with SCD have been very limited and included hydroxyurea, chronic blood transfusions, and bone marrow or stem cell transplantation. These therapies vary in their effectiveness and are associated with serious risks and tolerability issues [7]. In 2017, the US Food and Drug Administration (FDA) approved l-glutamine [8], the first new drug for SCD since hydroxyurea was approved for adults in 1998. Two additional therapies, voxelotor [9] and crizanlizumab [10], received FDA approval in 2019. In addition, numerous novel agents and gene therapies for SCD are currently in clinical trials [11].

With this long overdue increase in the pipeline for SCD therapies, the time is ripe to ensure a robust body of evidence on the safety, efficacy, and effectiveness of these new interventions is available for regulatory approval, health technology assessment, market access (coverage and reimbursement), and individual treatment decisions. Harmonization of the outcomes selected across interventional trials enables optimal post-trial appraisal and decision making through valid pooled analyses and indirect comparisons. It is critical to ensure that selected outcomes reflect meaningful benefits of therapy for patients and are useful for decisions faced by regulators, payers, and other stakeholders.

This can be accomplished by employing a structured, multi-stakeholder consensus process to develop a core outcome set (COS). A COS is an agreed standard set of outcomes that should be measured and reported, at a minimum, across clinical trials in a specific disease area [12]. In this paper, we describe coreSCD, an initiative aimed at developing a core set of outcomes for use in clinical trials of SCD interventions.

There are many factors involved in selecting outcomes for a clinical trial including phase, sample size, duration, and the cost and/or level of difficulty involved in measuring different outcomes. The COS development process begins by considering what should be measured separately from how selected outcomes are measured. However, the feasibility and burden of capturing specific outcomes is a key consideration in trial design and there is always a tradeoff between the amount and complexity of data collection and the speed and efficiency of trial completion.

For serious, rare diseases with few treatment options such as SCD, limiting unnecessary barriers to regulatory approval and market access is also an important consideration. Under the FDA Accelerated Approval program, products may be approved based on surrogate endpoints that are deemed reasonably likely to predict clinical benefit with the expectation that clinical benefit will be verified in post-approval confirmatory trials [13]. In developing a COS under these circumstances, the core set may include outcomes that are reasonable to collect in both Phase 3 (pivotal) and Phase 4 (post-regulatory) trials, as well as outcomes that may only be feasible to include in Phase 4 trials (e.g. those that require a longer time period to adequately assess). Ultimately, the goal of a COS is to ensure that a consistent body of evidence is available to inform decisions by regulators, payers, providers and patients and to improve patient access to effective, high-value treatments. Decisions about how a specific COS should be developed and implemented must be made with this in mind.

### coreSCD scope

Defining scope is a critical, and often challenging, first step in developing a core outcome set [14]. In addition to specifying the health condition to which the COS applies, decisions must be made about the target population (everyone with a given condition or a subset of the population), types of interventions (e.g. drugs or gene therapies), and categories of outcomes (e.g. clinical, functioning, resource utilization, biomarkers).

Several decisions about the scope of coreSCD were made at the beginning of the COS development process with the goal of creating a COS for trials of SCD interventions that is as broadly applicable as possible while also being feasible to implement. It is important to keep in mind that a COS is intended to be a minimal set of cross-cutting outcomes that in no way represents the full range of outcomes that might be important to include in any given trial. In addition, core outcomes may be included as secondary or exploratory rather than primary endpoints.

#### Population

The targeted age group was a key consideration in determining the scope of this COS. Given the life-long impact of SCD, our goal was to create a core set that is applicable across age groups. Although the relative importance of specific outcomes may vary by age, it was agreed that most outcomes have relevance across age groups and that differences often emerge in considering how certain outcomes are measured (e.g., pain is a critical outcome for all ages, but is assessed differently for children). Outcomes that are exclusively relevant for a specific age group were not considered eligible for the core set.

#### Interventions

The current pipeline of drug and gene therapies for SCD includes curative options (e.g. gene therapy) and other interventions intended to prevent or reduce the occurrence of SCD-related symptoms and complications (e.g. hydroxyurea, voxelotor, and crizanlizumab). For this project, we collectively termed these interventions “disease-modifying therapies” as they collectively seek to improve long term outcomes. While there are additional outcomes that may only be relevant to trials of gene therapies, many important outcomes will apply to both types of interventions.

Another subcategory of therapies designed to reduce the duration of SCD crises can be described as “acute interventions.” Trials of acute interventions are typically shorter in duration, not focused on longer term outcomes, and may require a more limited set of outcomes. Therefore, separate core sets were developed for trials of acute interventions and disease-modifying therapies.

#### Outcomes

Core outcome sets can encompass a wide range of outcome types, making COS useful for decision making across the product life cycle. Typical categories include physiological/clinical outcomes, biomarkers, functioning/quality of life, resource utilization, and survival/mortality. Because regulatory policies require documentation of side effects and adverse events, we opted not to include these outcomes as part of the consensus process. This is not to say that these outcomes are less important, but rather that their inclusion is typically not at the discretion of the researchers to whom COS recommendations are directed.

## Methods

### Overview

CoreSCD utilized a modified Delphi method adapted from the standards recommended by the Core Outcome Measures in Effectiveness Trials (COMET) Initiative and is registered in the COMET database (https://comet-initiative.org/studies/details/1284) [14]. The project took place between February 2019 and March 2020 and included four stages: 1) recruitment and orientation of a multi-stakeholder voting panel; 2) background research including identification of outcomes for consideration; 3) Delphi surveys to condense and prioritize the outcome list; and 4) an in-person consensus meeting to discuss outcomes remaining after the first two rounds of voting.

As a first step, we convened an 11-member Advisory Committee that included SCD clinicians and researchers with diverse areas of expertise (including adult and pediatric care and experience in acute care settings) as well as patient representatives. The Committee met regularly throughout the project period to participate in key decisions related to the project scope, initial list of candidate outcomes, outcome definitions, composition of the Delphi panel, survey design, interpretation of survey results, and dissemination.

### Multi-stakeholder Delphi panel

#### Recruitment

Patients, clinicians, researchers, payers, representatives from health technology assessment (HTA) organizations, regulators, and industry representatives were invited to participate in the Delphi panel. Our goal was to include clinicians with diverse expertise (e.g. pediatric and adult providers) and patients that were diverse with regard to gender, age, and geographic location. Recruitment of clinicians and patients was limited to the US due to the expense associated with bringing participants from other countries to the in-person meeting. We invited individuals with SCD over the age of 18, as well as family members of people with SCD, with the goal that patient representatives comprise at least 20% of the panel. Two representatives from each participating life science company with a SCD product in the pipeline were invited. Although the exact size of the Delphi Panel was not predetermined, our approach to COS development typically involves a panel size of 40–60 participants. Potential panel members were identified through a variety of sources including recommendations from Advisory Committee members, online searches of advocacy organizations and conference attendees, and in-person networking at relevant conferences. Potential participants were contacted by email and invited to participate in a phone call to learn more about the project and assess their interest in participating. All panel members (with the exception of industry representatives) were reimbursed for expenses associated with attending the in-person meeting and patient participants received a $500 honorarium as compensation for their time and effort. The study protocol was submitted to Advarra and determined to be exempt from Institutional Review Board (IRB) oversight. Although participants were fully informed about the project goals, procedures, and expectations, formal informed consent was not required for participation.

#### Orientation and training

Providing appropriate orientation and training is essential for any multi-stakeholder research initiative and this is particularly true for COS development projects. Patient participation in COS development has the advantage of incorporating patient values “upstream” in the development of new treatments, but this requires some understanding of the complex process by which outcome selection in clinical trial design has an impact on the evidence available for “downstream” decision making [15]. To ensure patients and other stakeholders were adequately prepared to participate, we conducted a series of one-hour webinars in advance of the first round of the Delphi survey that included: 1) kickoff and general orientation to project goals and methods; 2) introduction to clinical trial phases, design, and outcomes; 3) introduction to core outcome sets and the Delphi process; and 4) review of the format, content, and instructions for completing the Delphi surveys.

### Identification of preliminary list of outcomes

The coreSCD team developed an initial list of candidate outcomes from a targeted literature review that included searches of 1) clinical trial records on clinicaltrials.gov; 2) systematic reviews related to SCD identified in the Cochrane Library; and 3) articles related to therapeutic development in SCD in the journals *Blood* and *Blood Advances*. Our search was limited to randomized controlled trials of SCD interventions without regard to the age of the study population.

Studies involving non-pharmacological treatment (e.g. behavioral interventions) and studies on treating the side effects of interventions (e.g. chelation therapy for preventing iron overload from blood transfusion) were excluded. We limited our search to the past 5 years (January 2013–January 2019) based on the assumption that an outcome that was last used more than 5 years ago has not persisted as important to measure in this disease area.

In addition to including outcomes that have been used in recent trials, we sought to identify outcomes that are important to patients and other stakeholders that may not have been included in trials to date. We relied on two key sources to accomplish this objective: 1) a public meeting on sickle cell disease patient-focused drug development hosted by the FDA in February 2014 [16] and a SCD clinical endpoints workshop hosed by the FDA and the American Society for Hematology (ASH) in October 2018 [17, 18]. Outcomes identified as important during either of these events that were not already included in our initial list of candidate outcomes were added. In addition, members of both the Advisory Committee and the Delphi Panel were invited to suggest additional outcomes in advance of or following the first round of the Delphi survey.

Members of the Advisory Committee reviewed the initial list of outcomes and provided input regarding elimination of duplicate outcomes (i.e. those with similar names representing the same outcome) and categorization of outcomes into domains.

### Consensus process

#### Delphi voting

Three rounds of online voting were conducted using Qualtrics software (Qualtrics, Provo, UT). The survey was divided into two sections for trials of disease modifying therapies and trials of acute interventions. Delphi panelists were asked to rate each outcome on a scale of 1–9 where: 1–3 = *The outcome should not be included in the core outcome set*, 4–6 = *The outcome is important, but not critical to include in the core outcome set*, and 7–9 = *The outcome is critical to include in the core outcome set*. A *Don’t Know/No Opinion* option was included for each outcome with panelists instructed to select this option only if they had no opinion, were unsure of the importance of the outcome, or otherwise had uncertainties that would make it impossible for them to form an opinion.

Criteria for retaining or eliminating an outcome after each round of voting are summarized in Table 1. The “patient-important” criterion was applied in the first two rounds of voting to ensure that outcomes considered critical by patients were not eliminated prior to discussion at the in-person meeting. The patient-important criterion was dropped in the final round and only outcomes that reached high consensus for the full voting panel were included in the final COS.

**Table 1:** Consensus Rules

**High Consensus**	≥70% of all voters rated the outcome 7, 8, or 9
**Patient-Important**	< 70% of all voters rated the outcome 7, 8 or 9, BUT ≥70% of the patient group rated the outcome 7, 8, or 9
**Eliminate**	< 70% of all voters rated the outcome 7, 8 or 9, AND < 70% of the patient group rated the outcome 7, 8, or 9

After each of the first two rounds of voting, the coreSCD team analyzed the responses and provided participants a summary table of the results for each outcome overall and stratified by stakeholder group. Each voter also received an individual report that included their own rating of each outcome alongside the mean rating for the full panel and for each stakeholder group. Individual responses were highlighted if they deviated by two or more points from the average for all voters.

#### In-person meeting

A full day in-person meeting was held after the second round of voting, in October 2019, in Baltimore, Maryland. The meeting was scheduled to coincide with the SCDAA Annual Convention and was held in the same venue for the convenience of coreSCD participants who also planned to attend the Convention. All members of the Delphi Panel and the Advisory Committee were invited to participate and, with the exception of industry representatives, were reimbursed for their travel expenses.

The primary objective of the meeting was to provide a forum for all participants to share their opinions on the remaining candidate outcomes and to gain a better understanding of the views of others. Due to time constraints, the meeting focused on outcomes for trials of disease-modifying therapies with a web conference held after the meeting to discuss outcomes for trials of acute interventions. The meeting was structured to maximize discussion and ensure everyone in attendance was able to participate fully. Table assignments were made in advance so that each small group of approximately eight participants included at least one patient representative, clinician, payer or health technology assessor, and industry representative. The agenda included small group discussions on subsets of related outcomes interspersed with full group discussions led by an expert facilitator. The meeting was audio-recorded and transcribed to enable full capture of the points raised. A summary of key points was provided to Delphi panelists to inform their final round of voting (See Additional file 1).

## Results

### Participants

The 44-member Delphi Panel included 11 patients/advocates, 10 clinicians/researchers, 9 payers/health technology assessors, 3 government/funding agency representatives (FDA, Centers for Disease Control and Prevention [CDC], and Patient-Centered Outcomes Research Institute [PCORI]), and 11 industry representatives. All of the patients/advocates and clincians/researchers were from the US. Payers/HTA were primarily from the US with the exception of 1 HTA representative from Italy, 2 from the UK, and 1 from France. 42 (95%) of the panelists completed all three rounds of the online Delphi survey (1 industry representative dropped out prior to the first round of voting and 1 patient was unable to complete all rounds due to health issues).

### Delphi voting

**Fig. 1:**
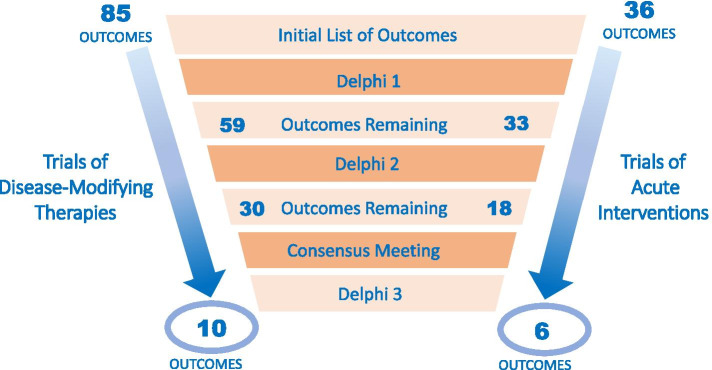
Delphi Results

After review by the Advisory Committee to exclude outcomes that did not meet criteria for inclusion and eliminate redundancies, the list of outcomes for the first round of Delphi voting included 85 outcomes for trials of disease-modifying therapies and 36 outcomes for trials of acute interventions (see Additional file 2 for list of outcomes). The results from each round of voting are summarized in Fig. 1.

An additional 15 outcomes for trials of disease-modifying therapies and 7 outcomes for trials of acute interventions were eliminated or combined with other outcomes based on discussion at the in-person meeting and subsequent approval by the Delphi panel. For example, the outcome originally labeled “vaso-occlusive crisis” was replaced with three outcomes for acute sickle cell pain frequency, duration, and intensity. (See Additional file 3 for full list of modifications).

Although biomarkers were included in early voting rounds, it was postulated at the in-person consensus meeting that, because the value of a specific biomarker is linked with the mechanism of action of an intervention, it is not feasible to include them in a core set applicable across interventions. The decision to remove biomarkers from the final round of voting was subsequently adopted. This is not to say that biomarkers are unimportant as clinical trial outcomes, but that their selection must be trial-specific.

### Final Core sets

After the final round of voting, 10 outcomes were retained for trials of disease-modifying therapies and 6 outcomes were retained for trials of acute interventions (Table 2; see Additional file 4 for definitions).

**Table 2:** Final Core Sets

Core Outcome Set for Clinical Trials of Disease-Modifying Therapies	Core Outcome Set for Clinical Trials of Acute Interventions
• Acute sickle cell pain frequency • Acute chest syndrome • Stroke or cerebrovascular accident • Neurocognitive function • Health-related quality of life • Frequency of hospitalization • Emergency department/acute care visit • Need for blood transfusion • Cause-specific survival/mortality • Event-free survival	• Acute sickle cell pain frequency • Acute chest syndrome • Ability to return to usual activities • Frequency of hospitalization • Emergency department/acute care visit • Cause-specific survival/mortality

## Discussion

We employed a methodologically rigorous, transparent process to reach multi-stakeholder consensus on core sets of outcomes that are critical to include in clinical trials of disease-modifying therapies and acute interventions for SCD. The perspectives of those who rely on the evidence to make decisions, including patients, providers, health technology assessors, payers, and regulators, were incorporated at every step of the project. Meaningful engagement of patients and advocates is a fundamental aspect of our approach to COS development as evidenced by the significant number of individuals with SCD and their family members who participated in coreSCD, emphasis on orientation and training, and inclusion of a “patient-important” criterion in the first two rounds of the Delphi survey (see Clearfield et al., 2020 for a more detailed description of our patient-centered approach to COS development) [15].

This project builds on previous efforts to understand the treatment outcomes that are most important to individuals living with SCD and to harmonize outcome measurement across clinical trials for SCD interventions. The American Society of Hematology (ASH)/FDA SCD Clinical Endpoints Workshop convened seven expert panels in 2018 to develop important recommendations on appropriate outcomes, measures, and endpoints for SCD clinical trials [17, 18]. More recently, the National Heart, Lung, and Blood Institute (NHLBI) *Cure Sickle Cell Initiative* released the *CureSCi Common Data Elements* (CDE) to facilitate data collection on research studies of genetic therapies for SCD, including recommendations related to outcomes and endpoints [19]. As noted below results from coreSCD are consistent with ASH/FDA and CureSCi recommendations in many ways. However, key differences between the three initiatives should be noted. First, whereas both the ASH/FDA and CureSCi workgroups were comprised primarily of clinical experts with the addition of one or two patient representatives, the coreSCD Delphi Panel included roughly equivalent numbers of patient representatives (11), clinical experts (10), payers and health technology assessors (9), and industry representatives (11). As such, the coreSCD COSs represent the evidence priorities of a full range of decision makers. In addition, coreSCD is the only one of the three initiatives to use a structured consensus process with multiple rounds of anonymous voting and clear criteria for inclusion and exclusion of candidate outcomes with the goal of agreeing upon *minimal* sets of outcomes that can feasibly be included across all relevant SCD interventional trials. Finally, whereas CureSCi is focused exclusively on genetic therapies, both ASH/FDA and coreSCD considered a broader range of therapies and only coreSCD developed separate recommendations for trials of disease-modifying versus acute interventions.

Outcomes associated with pain were, not surprisingly, an important area of focus throughout this project. Six pain outcomes remained after the second round of voting and were discussed at the in-person meeting. Although pain interference/impact is an important treatment outcome, the group agreed that it should be considered as a component of health-related quality of life (HRQOL) and functioning. Pain outcomes in the final Delphi survey included frequency, intensity, and duration of acute sickle cell pain as well as chronic pain. Of these, only frequency of acute sickle cell pain met the criterion for inclusion in the final core set. Discussion at the in-person meeting focused on the measurement challenges associated with pain intensity, which may explain why it was not retained. Although pain duration was not included in the final core set, it came very close to meeting the criterion for inclusion in the COS for trials of acute interventions (69%) and may be an important outcome to include in certain trials. Chronic pain was also eliminated in the final round of voting, although it was rated more highly by patients (73%) than other stakeholder groups. Meeting discussion emphasized that chronic pain may result from a new disease complication (e.g. avascular necrosis) that is outside the treatment scope of the therapy under investigation. It was suggested that measurement of chronic pain is particularly important in trials of curative therapies initiated early in life.

SCD has a significant impact on the quality of life (QOL) and functioning of individuals living with the disease and the importance of incorporating patient-reported outcomes (PRO) in assessing new interventions is increasingly recognized. While HRQOL is represented by a single outcome in the final core set, the Delphi panel stressed that HRQOL assessments in SCD trials should encompass social, emotional, cognitive, and physical functioning, including pain interference/impact on daily activities and fatigue. This is consistent with ASH/FDA workshop and CureSCi recommendations, which also include specific recommendations regarding the measurement of HRQOL outcomes for both adults and children [17, 19]. Although comprehensive HRQOL was not included in the COS for trials of acute interventions, ability to return to usual activities is one aspect of functioning that was deemed essential for these types of trials.

In addition to cognitive functioning outcomes that are included in PRO instruments, coreSCD participants felt that objectively measured neurocognitive functioning is a critical outcome for SCD clinical trials. This is again consistent with ASH/FDA recommendations, which note that a proportion of affected individuals develop deficits in executive function, processing speed, working memory, and attention with or without a history of silent cerebral infarcts [17]. The CureSCi CDE also includes multiple measures of cognition which are categorized as supplemental (but often highly recommended) as opposed to core data elements [19].

A range of outcomes related to use of healthcare resources were included in the list of candidate outcomes. Frequency of hospitalization and frequency of emergency department or acute care visits were retained in both of the final core sets and for trials of disease-modifying therapies, need for blood transfusion was also retained. Other resource use outcomes that were eliminated during the final round of voting included length of hospital stay, intensive care unit (ICU) admission, and hospital readmission. Discussion of these three outcomes revealed that they are all highly dependent on the policies and procedures of individual hospitals, and therefore may not be reliable indicators of treatment effectiveness.

Sickle cell disease is a phenotypically complex and highly variable condition associated with a wide range of acute and chronic complications across the lifespan of individuals living with the disease. As such, there are many important treatment outcomes for trials of SCD interventions beyond those included in the final core outcome sets. For example, chronic kidney disease and cardiopulmonary dysfunction are common and life-threatening complications that, although not retained through the consensus process, are vitally important for longer-term Phase 4 trials. The need for better treatments to address these and other chronic complications was emphasized at the SCD patient-focused drug development meeting convened by the FDA in 2014 [16]. Pregnancy complications is another outcome that was not retained despite being considered critically important by stakeholders, especially individuals with SCD. The need for more data on the impact of SCD on reproductive health and women’s health more broadly was strongly emphasized at the in-person meeting.

Although developed specifically for clinical trials, the COS also highlights gaps in health services for SCD. Neurocognitive function is among the retained outcomes, but neurocognitive testing is usually available only in the context of research studies due to limited health insurance coverage. Cause-specific mortality is difficult to ascertain without detailed chart review, and death certificates are often filled out with incomplete information by a physician on call rather than the continuity physician. Health-related quality of life is not assessed routinely in SCD care and documentation of the “ability to return to usual activities” would be impossible to find in administrative datasets and inconsistent in manual chart review. Thus, the Delphi panel noted that pragmatic trials or comparison of a clinical trial cohort to standard of care using administrative datasets will not be possible until the COS are reported as standard of care for SCD.

A few potential limitations to this work should be noted. First, although representatives from Europe were included, the participants on the Delphi panel were largely from the US. Outcomes included in the COS, particularly those associated with resource utilization, must be reconsidered for any application outside of the US health care system. Second, we sought to recruit patient representatives who were diverse with respect to gender, age, and geographic location. While we were successful in those dimensions, we must point out that all of the patient and caregiver participants had previous advocacy experience. This may mean that we have not captured the views of the most severely affected patients or those who are otherwise marginalized. Many of these coreSCD participants maintain contact with patients across geographic and socioeconomic groups via their grassroots advocacy efforts and organizations. When contributing to deliberations, patients and caregivers commented based on their personal views and trends reported by numerous other individuals living with SCD. Therefore, by nominating patients and caregivers who regularly engage with hundreds and in some cases thousands of patients and caregivers, we attempted to maximize the diversity of input from these perspectives.

## Conclusions

The coreSCD COS represents outcomes considered most important for the evaluation of new SCD interventions across multiple stakeholder groups. Consistent use and reporting of the COS in SCD clinical trials can help to ensure that a robust body of evidence is available for regulatory, coverage and reimbursement, and treatment decisions. When new therapies are approved based on surrogate endpoints under accelerated approval guidelines, the COS should be considered for ongoing trials designed to confirm clinical benefit. While our process led to agreement on *what* should be measured across trials, additional work is needed to further specify *how* each outcome in the core set should be measured.

## Supplementary Information

**Additional file 1.** Discussion Summary. Summary of key points from in-person meeting discussion.

**Additional file 2.** coreSCD Candidate Outcomes. List of all outcomes included at the start of the consensus process.

**Additional file 3.** Proposed Changes to List of Candidate Outcomes for Final Delphi Round. Changes proposed during in-person discussion and subsequently endorsed by Delphi Panel.

**Additional file 4.** coreSCD Outcomes with Definitions. List of all outcomes included in the final core sets with definitions provided to participants.

## Data Availability

The protocol and datasets used during the current study are available from the corresponding author on reasonable request.

## Abbreviations

ASH: American Society of Hematology
CDC: Centers for Disease Control and Prevention
COMET: Core Outcome Measures in Effectiveness Trials
COS: Core Outcome Set
FDA: Food and Drug Administration
HRQOL: Health-Related Quality of Life
HTA: Heath Technology Assessment
ICU: Intensive Care Unit
PCORI: Patient Centered Outcomes Research Institute
PRO: Patient-Reported Outcome
QoL: Quality of Life
SCD: Sickle Cell Disease
SCDAA: Sickle Cell Disease Association of America
