# Supplementary material for: coreSCD: multi-stakeholder consensus on core outcomes for sickle cell disease clinical trials
Source: BMC Med Res Methodol. 2021 Oct 19;21:219. doi: 10.1186/s12874-021-01413-8 (PMC8524872; doi:10.1186/s12874-021-01413-8)
Supplement: Supplementary file 1 — Additional file 1. Discussion Summary. Summary of key points from in-person meeting discussion. [file 12874_2021_1413_MOESM1_ESM.pdf]

DISCUSSION SUMMARY

Part 1: Outcomes for Trials of Disease Modifying Therapies

October 9th In-Person Meeting

General Considerations

- In addition to developing separate core sets for trials of disease-modifying and acute interventions, we need to determine which outcomes are appropriate for **Phase 3 vs. Phase 4** trials (Phase 3 trials are shorter in duration and include fewer participants than Phase 4 trials)

- It is critical that the core sets be very small (e.g. fewer than 10 outcomes for Phase 4 and around 3 outcomes for Phase 3), but this does not limit the total number of outcomes that can be measured for any specific trial

- Outcomes that are not eliminated through the Delphi process can be assigned to one of **three categories**:
  - Outcomes that should be included in **all Phase 3 and Phase 4 trials** of disease-modifying therapies or acute interventions
  - Outcomes that should be included in **all Phase 4 trials**, but might not be appropriate or feasible for Phase 3 trials
  - Outcomes that are **critical for certain types of research**, but do not belong in a core set

Pain Outcomes

Round 2 Delphi Results	**Overall (N=44)**	**Patients/ Advocates (N=10)**	**Clinicians/ Researchers (N=10)**	**HTA/ Payers (N=9)**	**Govt. Agencies (N=4)**	**Industry (N=11)**
**Pain frequency**	**89%**	90%	90%	89%	75%	91%
**Pain intensity**	**84%**	90%	80%	89%	75%	82%
**Vaso-occulsive crisis**	**82%**	90%	70%	78%	75%	91%
**Pain duration**	**80%**	80%	90%	78%	50%	82%
**Pain interference /impact**	**79%**	80%	80%	88%	50%	82%
**Chronic pain***	**70%**	70%	60%	78%	75%	73%

- **Pain interference/impact**
  - General agreement that belongs in the functioning domain as an aspect of physical functioning
- **"Pain due to VOC"** (renamed because VOC is the cause and pain is the effect)
  - Suggested as primary pain outcome for the COS
  - Definition includes measurement of pain frequency, duration, and intensity
  - Discussed how VOC is determined - directly by patient or with input from clinician; in either case, requires education about the distinction between crisis pain and chronic pain
- **Chronic pain**
  - Might belong with pain interference
  - Can result from new disease process rather than sickling
  - Becomes more important with evolution of therapies (Phase 4, not Phase 3)

Neurocognitive Outcomes

Round 2 Delphi Results	Overall (N=44)	Patients/ Advocates (N=10)	Clinicians/ Researchers (N=10)	HTA/ Payers (N=9)	Govt. Agencies (N=4)	Industry (N=11)
**Stroke/cerebrovascular accident**	**95%**	90%	100%	100%	75%	100%
**Silent cerebral infarcts**	**88%**	78%	90%	75%	100%	100%
**Transient ischemic attack**	**79%**	62%	90%	78%	75%	82%

- Outcomes in this category more appropriately labeled as *neurocognitive complications* that can have an impact on *neurocognitive function*
- Deficits in neurocognitive function seen in children without known complications; but formal neurocognitive testing takes time and an easier screening test has not be found
- For Phase 3 and Phase 4 trials, neurocognitive function would not be considered as an endpoint; but for children, impact of drug to normalize TCD is very important
- Stroke is very important from a resource utilization standpoint
- Big category is important, but which you look at depends on type of study

Fatigue

Round 2 Delphi Results	Overall (N=44)	Patients/ Advocates (N=10)	Clinicians/ Researchers (N=10)	HTA/ Payers (N=9)	Govt. Agencies (N=4)	Industry (N=11)
**Fatigue**	**84%**	90%	78%	67%	75%	100%

- Fatigue is clinically meaningful and of very high importance to patients; number one concern for many patients because they don't deal with pain as often
- Multifactorial symptom that includes physical and mental fatigue and tiredness; can be tied to acute and chronic pain, cognitive functioning, economic functioning, sleep, depression, etc.
- Very hard to study impact on fatigue in a single, short-term trial, so might not work as a core outcome
- May fit better under functioning and already included in some functioning scales
- For payers, not meaningful unless in a functioning context- they're interested in putting people back to school and work and not ready to pay for fatigue itself
- May be more appropriate as an exploratory, rather than primary or key secondary, endpoint; can be used to inform patients even if not on the label, which is important because fatigue is linked to compliance (e.g. hydroxyurea vs. l-glutamine)
- Need to define and figure out best way to measure
- Have to consider tradeoff with other potential treatment effects

Other Physiological/Clinical Outcomes

Round 2 Delphi Results	Overall (N=44)	Patients/ Advocates (N=10)	Clinicians/ Researchers (N=10)	HTA/ Payers (N=9)	Govt. Agencies (N=4)	Industry (N=11)
**Sickle cell nephropathy**	**74%**	67%	60%	100%	50%	82%
**Acute kidney injury**	62%	**75%**	40%	62%	75%	70%
**Acute chest syndrome**	**84%**	80%	100%	78%	50%	91%
**Pulmonary hypertension**	**70%**	60%	80%	67%	50%	82%
**Pregnancy complications**	66%	**78%**	70%	75%	50%	50%

- **Acute kidney injury**
  - This is a complication of treatment and a safety measure, therefore should not be included in core set (safety measures related to new treatments will be a regulatory requirement and therefore no need to reach agreement through multi-stakeholder consensus process)
- **Pregnancy complications**
  - Very important issue that needs further study, but not appropriate as a clinical trial outcome at this point because we don't know enough about it
- **Acute chest syndrome, sickle cell nephropathy, and pulmonary hypertension**
  - All associated with high mortality so important to address
  - ACS is good for Phase 3 and 4 trials, but nephropathy and pulmonary hypertension more appropriate for Phase 4
  - Proposal to use "end organ damage" as a category that encompasses these three

Biomarkers

Round 2 Delphi Results	Overall (N=44)	Patients/ Advocates (N=10)	Clinicians/ Researchers (N=10)	HTA/ Payers (N=9)	Govt. Agencies (N=4)	Industry (N=11)
**Level of hemoglobin**	**92%**	100%	100%	86%	100%	82%
**Level of fetal hemoglobin**	69%	**86%**	80%	60%	67%	55%
**Level of sickle hemoglobin**	63%	**88%**	70%	67%	67%	36%
**Hemolysis**	**73%**	86%	67%	57%	67%	82%
**Change in hematocrit**	**71%**	40%	78%	86%	100%	60%
**Oxygen % saturation**	55%	**100%**	44%	20%	50%	50%

- Suggestion that these biomarkers should not be part of a core set because they are treatment-specific
- From HTA perspective, biomarkers not sufficient without strong correlation to clinical endpoints
- Patients concerned about overreliance (by payers) on hemoglobin as an indicator of disease severity
- Noted that hemoglobin is ubiquitously reported in the "real world"

Round 2 Delphi Results	Overall (N=44)	Patients/ Advocates (N=10)	Clinicians/ Researchers (N=10)	HTA/ Payers (N=9)	Govt. Agencies (N=4)	Industry (N=11)
**Cardiac Function**	**89%**	100%	78%	100%	100%	100%
**Kidney Function**	**86%**	100%	89%	86%	67%	80%
**Transcranial Doppler Velocities**	**86%**	86%	90%	50%	100%	100%
**Lung Function**	**72%**	100%	78%	57%	67%	60%
**Liver Function**	54%	**83%**	67%	43%	67%	30%
**Splenic Function**	42%	**86%**	30%	50%	67%	10%

Functioning Outcomes

Round 2 Delphi Results	Overall (N=44)	Patients/ Advocates (N=10)	Clinicians/ Researchers (N=10)	HTA/ Payers (N=9)	Govt. Agencies (N=4)	Industry (N=11)
**Cognitive function**	**93%**	90%	100%	89%	75%	100%
**Physical function**	**84%**	70%	90%	78%	75%	100%
**Health-related (global) quality of life**	**89%**	80%	90%	89%	100%	91%
**Depression**	**66%**	80%	60%	67%	25%	73%
**Anxiety**	50%	**80%**	30%	44%	25%	55%
**Future orientation***	53%	**89%**	25%	33%	50%	57%
**Missed days at school/work**	77%	**60%**	80%	78%	50%	100%
**Patient satisfaction w/treatment**	70%	**70%**	60%	56%	75%	91%

- Missed days of school/work important as both an economic outcome and an aspect of social functioning
- Depression and anxiety difficult – are they being looked at as a direct result of treatment? They're relevant, but don't stand out as much in SCD population as physical and cognitive functioning
- There are conflicting priorities with regard to generic vs. disease-specific HRQOL instruments – generic important from health economics point of view (and in some countries is required) so can compare SCD to other diseases, but trial sponsors want a measure that is as specific and sensitive as possible
- In the US, HRQOL is considered a research tool
- Point made that having everyone use the same measure is more important than having a measure that includes everything; ASCQ-Me may be suitable for this purpose, but no sure it measures everything adequately
- Also need to keep in mind that the more you add the less sensitive the tool becomes, and the more burden on patients to complete
- Economic burden suggested as additional HRQOL outcome

Resource Use

Round 2 Delphi Results	Overall (N=44)	Patients/ Advocates (N=10)	Clinicians/ Researchers (N=10)	HTA/ Payers (N=9)	Govt. Agencies (N=4)	Industry (N=11)
**Emergency dept visit**	**84%**	80%	80%	78%	100%	91%
**Acute care visit**	**77%**	70%	80%	67%	100%	82%
**Freq of hospitalization**	**84%**	80%	80%	89%	75%	91%
**Freq of ICU admission**	**70%**	70%	70%	78%	50%	73%
**Length of hospital stay**	**73%**	80%	60%	78%	50%	82%
**Hospital readmission**	**82%**	80%	80%	89%	50%	91%
**Need for blood transfusion**	**75%**	60%	80%	78%	50%	91%

- **Emergency department visits and frequency of hospitalization**
  - Consistently ranked as most important
  - Highest cost to system and greatest burden to patients
- **Acute care visit**
  - Much lower burden for patients, but not an option for everyone
  - May need to be combined with ED visit
- **Need for blood transfusion**
  - Also considered important by many in the room
  - Argument against including: it's the reason for the blood transfusion that's important and this should be captured elsewhere
  - Counterargument: it's still important as a cost to system and burden to patients
- **Length of hospital stay and ICU admissions**
  - Dependent on health system rules, decision-making by specific doctor, etc., so not as useful for measuring impact of treatment
- **Hospital readmission**
  - May not be a good outcome to determine the impact of an intervention – unpredictable and multifactorial
  - More a marker of hospital quality than effectiveness of intervention
  - Argument for including based on paper describing readmission rates for SCD patients; readmission within 2 weeks considered a continuation of previous admission; most common reasons for readmission were poor treatment and premature discharge
- **Indirect costs**
  - Include missed days of school or work for patients and their caregivers
  - Some countries take these into account in health technology assessment

Mortality and Survival

Round 2 Delphi Results	Overall (N=44)	Patients/ Advocates (N=10)	Clinicians/ Researchers (N=10)	HTA/ Payers (N=9)	Govt. Agencies (N=4)	Industry (N=11)
**Cause-specific survival/mortality**	**84%**	90%	100%	89%	75%	64%
**Event-free survival**	**79%**	70%	100%	89%	75%	60%
**All-cause survival/mortality**	**75%**	90%	100%	67%	50%	55%

- **Event-free survival**
  - Useful for assessing quality of life years gained for a particular intervention
- **Cause-specific mortality**
  - Of greatest interest
  - Includes any cause directly related to SCD
  - Most frequent (in order) include acute chest syndrome, VOC, infection, and chronic renal failure
- **All-cause mortality**
  - Can be useful to look at in addition to cause-specific because might identify other causes that are higher in SCD than in the general population
- Suggested that these outcomes not be part of the core set

Part 2: Outcomes for Trials of Acute Interventions

November 6th Web Conference

Definition

- Trials of acute interventions:
  - Involve treatments for acute complications such as vaso-occlusive crisis that require rapid intervention
  - Goal is to alleviate symptoms and lessen the risk of life-threatening complications
  - Typically shorter in duration than trials for disease-modifying therapies (days/weeks rather than months/years)
  - Often conducted in acute care setting such as a hospital

Pain Outcomes

	Overall (N=44)	Patients/ Advocates (N=10)	Clinicians/ Researchers (N=10)	HTA/ Payers (N=9)	Govt. Agencies (N=4)	Industry (N=11)
**Vaso-occlusive crisis**	**91%**	80%	90%	100%	100%	91%
**Pain intensity**	**89%**	90%	70%	89%	100%	100%
**Pain duration**	**80%**	80%	60%	78%	75%	100%
**Pain frequency**	**80%**	70%	70%	89%	75%	91%
**Pain interference /impact**	**79%**	78%	70%	75%	75%	91%
**Opioid use**	66%	**70%**	60%	44%	75%	82%

- **"Pain due to VOC"** was suggested as primary pain outcome for the COS at in-person meeting, but it was recommended that this be changed to **"acute pain episode"**
  - Until there are biomarkers or some other means of identifying VOC, and in the absence of an agreed-upon definition of VOC, outcome should be independent of mechanism
- **Pain frequency, intensity, and duration** are all included in definition of **acute pain episode** but should be kept as 3 distinct outcomes
  - Intensity and duration are difficult to measure, particularly outside of acute setting
  - Pain frequency more important for trials of disease-modifying therapies, but intensity and duration important for trials of acute interventions
  - Composite endpoints are problematic from clinical trial viewpoint, so if in doubt should avoid "lumping"
- **Opioid use** should be retained
  - If drugs are developed for acute pain that are improvement over opioids, opioid use would be important endpoint
  - Also noted that opioid use could be an important endpoint for trials of chronic pain interventions
- Discussion highlights difference in understanding – people administering trials view pain frequency, intensity, and duration as a concept and attempt to measure without fully understanding the experience of patients

Other Physiological/Clinical Outcomes

	Overall (N=44)	Patients/ Advocates (N=10)	Clinicians/ Researchers (N=10)	HTA/ Payers (N=9)	Govt. Agencies (N=4)	Industry (N=11)
**Acute chest syndrome**	**91%**	90%	100%	89%	50%	100%
**Stroke/cerebrovascular accident**	**89%**	100%	100%	67%	50%	100%
**Venous thromboembolism**	**71%**	71%	60%	78%	75%	73%
**Silent cerebral infarcts**	56%	**78%**	60%	22%	50%	64%
**Fatigue**	39%	**70%**	30%	44%	50%	9%

- **Venus thromboembolism** is an indirect outcome linked to heavy medication during acute crisis, therefore not important to include in core set
- **Stroke/CVA** would become important if there are interventions in the future
- Noted that heart failure not included (eliminated based on earlier voting) but considered important. Points to lack of basic SCD research to inform clinical trials.
- **Fatigue**
  - Important reflection of patient experience but difficult to measure
  - Related to **return to usual activities**, which is an important outcome for trials of acute interventions
  - Patients report notable increase in fatigue leading up to and during acute pain crisis
  - Noted lack of understanding about severity and impact of fatigue on people with SCD

Biomarkers

	Overall (N=44)	Patients/ Advocates (N=10)	Clinicians/ Researchers (N=10)	HTA/ Payers (N=9)	Govt. Agencies (N=4)	Industry (N=11)
**Level of hemoglobin**	**89%**	86%	90%	100%	100%	78%
**Hemolysis**	**77%**	86%	67%	86%	100%	67%
**Oxygen % saturation**	**85%**	100%	89%	57%	100%	89%
**Kidney function**	**76%**	86%	67%	86%	50%	78%
**Lung Function**	**74%**	86%	78%	71%	50%	67%

Functioning Outcomes

	Overall (N=44)	Patients/ Advocates (N=10)	Clinicians/ Researchers (N=10)	HTA/ Payers (N=9)	Govt. Agencies (N=4)	Industry (N=11)
**Ability to return to usual activities**	**86%**	90%	90%	78%	50%	100%
**Patient satisfaction with treatment**	**73%**	70%	90%	33%	50%	100%

Resource Use

	Overall (N=44)	Patients/ Advocates (N=10)	Clinicians/ Researchers (N=10)	HTA/ Payers (N=9)	Govt. Agencies (N=4)	Industry (N=11)
**Emergency dept visit**	**82%**	90%	80%	78%	75%	82%
**Freq of hospitalization**	**82%**	80%	90%	89%	25%	91%
**Freq of ICU admission**	**75%**	70%	70%	78%	75%	82%
**Length of hospital stay**	**75%**	80%	60%	67%	50%	100%
**Hospital readmission**	**73%**	90%	40%	78%	50%	91%
**Need for blood transfusion**	**70%**	50%	80%	78%	50%	82%

- **Frequency of ICU admission**
  - Depends on setting (e.g. community-based hospital may elevate to ICU more readily than tertiary referral center)
- **Length of hospital stay**
  - Important when economics of novel therapies are examined by third parties
  - Should not be compared to a perceived average, but support looking at change for individual person
  - Most studies look at median length of stay to take into account "outliers"
- **Hospital readmission**
  - Important if looking at whether intervention makes it more or less likely for an acute episode to relapse or persist
- **Need for blood transfusion**
  - Not a useful outcome because the reasons are varied

Mortality and Survival

	Overall (N=44)	Patients/ Advocates (N=10)	Clinicians/ Researchers (N=10)	HTA/ Payers (N=9)	Govt. Agencies (N=4)	Industry (N=11)
**Cause-specific survival/mortality**	**86%**	90%	100%	89%	75%	73%
