# Supplementary material for: coreSCD: multi-stakeholder consensus on core outcomes for sickle cell disease clinical trials
Source: BMC Med Res Methodol. 2021 Oct 19;21:219. doi: 10.1186/s12874-021-01413-8 (PMC8524872; doi:10.1186/s12874-021-01413-8)
Supplement: Supplementary file 2 — Additional file 2. coreSCD Candidate Outcomes. List of all outcomes included at the start of the consensus process. [file 12874_2021_1413_MOESM2_ESM.pdf]

coreSCD Candidate Outcomes

Outcomes for Trials of Disease-Modifying Therapies (N=85)

**Physiological/Clinical**	**Functioning (cont.)**
Heart failure	Work and career performance
Retinopathy	School performance
Hearing loss	Health-related (global) quality of life
Fatigue	Patient satisfaction with treatment
Sleep interference	Perceived burden of treatment
Underweight	**Resource Use**
Splenic sequestration	Frequency of hospitalization
Hepatic sequestration	Length of hospital stay
Avascular necrosis	Hospital readmission
Gout	Frequency of ICU admission
Stroke or cerebrovascular accident (CVA)	Length of ICU stay
Silent cerebral infarcts	Emergency department (ED)
Transient ischemic attack (TIA)	Acute / urgent care visit
Brain volume	Need for blood transfusion
Brain aneurysm	**Morality/Survival**
Peripheral neuropathy	All-cause survival / mortality
Pain frequency	Cause-specific survival / mortality
Pain intensity	Event-free survival
Pain-relieving medication use	**Biomarkers/Biochemical Response**
Pain duration	Level of hemoglobin (Hb)
Pain interference and pain impact	Change in hematocrit (Hct)
Vaso-occulsive crisis (VOC)	Change in mean corpuscular volume (MCV)
Sickle cell neuropathy	Level of bilirubin
Need for dialysis	Level of lactate dehydrogenase (LDH)
Priapism	Reticulocyte count
Sexual function / dysfunction	Hemolysis
Sterility and infertility	Level of sickle hemoglobin (HbS)
Pregnancy complications	Level of fetal hemoglobin (HbF)
Acute chest syndrome	Platelet count
Pulmonary hypertension	Level of C reactive protein (CRP)
Leg ulceration	Change in the proportion of(DRBC)
Venous thromboembolism	Level of erythropoietin (EPO)
**Functioning**	Oxygen percent saturation (SAO2)
Functional exercise capacity	RBC alloantibodies
Physical functioning	Soluble adhesion molecules
Cognitive function	Liver function
Difficulty concentrating	Splenic function
Processing speed	Kidney function
Educational performance	Lung function
Anxiety	Cardiac function
Depression	Brain natriuretic peptide
Concerns about future health	Transcranial Doppler Velocities
Romantic relationships	MRI cerebral blood flow
Missed days at school / work	Oxygen extraction fraction
Families ability to work or make plans	

Outcomes for Trials of Acute Interventions (N=36)

**Physiological/Clinical**	**Resource Use**
Fatigue	Frequency of hospitalization
Sleep interference	Length of hospital stay
Splenic sequestration	Hospital readmission
Hepatic sequestration	Frequency of ICU admission
Stroke or cerebrovascular accident (CVA)	Length of ICU stay
Silent cerebral infarcts	Emergency department (ED)
Transient ischemic attack (TIA)	Acute / urgent care visit
Brain aneurysm	Need for blood transfusion
Pain frequency	**Biomarkers/Biochemical Response**
Pain intensity	Level of hemoglobin (Hb)
Pain-relieving medication use	Change in hematocrit (Hct)
Pain duration	Level of indirect bilirubin
Pain interference and pain impact	Level of lactate dehydrogenase (LDH)
Vaso-occulsive crisis (VOC)	Reticulocyte count
Acute chest syndrome	Hemolysis
Venous thromboembolism	Oxygen percent saturation (SAO2)
**Functioning**	Soluble adhesion molecules
Patient satisfaction with treatment	Kidney function
**Morality/Survival**	Lung function
Cause-specific survival / mortality	
