# Supplementary material for: coreSCD: multi-stakeholder consensus on core outcomes for sickle cell disease clinical trials
Source: BMC Med Res Methodol. 2021 Oct 19;21:219. doi: 10.1186/s12874-021-01413-8 (PMC8524872; doi:10.1186/s12874-021-01413-8)
Supplement: Supplementary file 3 — Additional file 3. Proposed Changes to List of Candidate Outcomes for Final Delphi Round. Changes proposed during in-person discussion and subsequently endorsed by Delphi Panel. [file 12874_2021_1413_MOESM3_ESM.pdf]

Proposed Changes to List of Candidate Outcomes for Final Delphi Round*

<table>
<tr><th>DECISION</th><th colspan="2">OUTCOMES (as included in Round 2 Delphi survey)</th><th>RATIONALE</th></tr>
<tr><td rowspan="10">Remove from consideration for core set

(although recommendations regarding any of these outcomes might still be part of the reporting from this project)</td><td colspan="2">Acute kidney injury</td><td>Complication of treatment that would be considered as a safety measure</td></tr>
<tr><td colspan="2">Pregnancy complications</td><td>Very important topic that needs further study, but not enough known at this point to include as a clinical trial outcome</td></tr>
<tr><td colspan="2">Venous thromboembolism</td><td>Indirect outcome linked to heavy medication during acute pain crisis</td></tr>
<tr><td colspan="2">Transcranial doppler velocities</td><td>Appropriate for pediatric but not adult trials</td></tr>
<tr><td colspan="2">Level of hemoglobin</td><td rowspan="6">There is substantial agreement that the relevance of each of these blood biomarkers is dependent on the mechanism of action of the specific intervention, and therefore not appropriate for a core set of outcomes relevant across interventions</td></tr>
<tr><td colspan="2">Level of fetal hemoglobin</td></tr>
<tr><td colspan="2">Level of sickle hemoglobin</td></tr>
<tr><td colspan="2">Hemolysis</td></tr>
<tr><td colspan="2">Change in hematocrit</td></tr>
<tr><td colspan="2">Oxygen % saturation</td></tr>
<tr><td rowspan="2">Move to different domain</td><td colspan="2">Pain interference/impact</td><td rowspan="2">These were originally included under physiological/clinical outcomes, but there is agreement that both should be considered aspects of functioning with the recommendation that they be measured as part of HRQOL</td></tr>
<tr><td colspan="2">Fatigue</td></tr>
<tr><td rowspan="2">Combine</td><td colspan="2">Sickle cell nephropathy <u>and</u> kidney function</td><td>A diagnosis of nephropathy occurs along the spectrum of kidney function and is based on the same types of measures</td></tr>
<tr><td colspan="2">Emergency department visit <u>and</u> acute care visit</td><td>Whether an individual with SCD goes to an emergency department or acute care center for a pain crisis is largely dependent on accessibility</td></tr>
<tr><td rowspan="4">Rename/redefine</td><td>VOC</td><td>(replaced by the following outcomes)</td><td rowspan="4">Goal is to capture experience of acute severe pain directly related to sickle cell that may or may not result in an individual seeking medical care.</td></tr>
<tr><td>Pain frequency</td><td>Frequency of acute sickle cell pain</td></tr>
<tr><td>Pain intensity</td><td>Intensity of acute sickle cell pain</td></tr>
<tr><td>Pain duration</td><td>Duration of acute sickle cell pain</td></tr>
</table>

***As discussed at in-person consensus meeting and subsequently endorsed by Delphi Panel**
