# Supplementary material for: coreSCD: multi-stakeholder consensus on core outcomes for sickle cell disease clinical trials
Source: BMC Med Res Methodol. 2021 Oct 19;21:219. doi: 10.1186/s12874-021-01413-8 (PMC8524872; doi:10.1186/s12874-021-01413-8)
Supplement: Supplementary file 4 — Additional file 4. coreSCD Outcomes with Definitions. List of all outcomes included in the final core sets with definitions provided to participants. [file 12874_2021_1413_MOESM4_ESM.pdf]

coreSCD Outcomes with Definitions

OUTCOME	DEFINITION
**Acute sickle cell pain- frequency**	How often the pain is experienced in a given time period. Acute sickle cell pain is pain directly related to sickle cell disease that is usually associated with vaso-occlusive crisis and may or may not result in an individual seeking medical care.
**Stroke or cerebrovascular accident (CVA)**	A traumatic complication of sickle cell disease, including ischemic and hemorrhagic stroke that is associated with intimal disease of the large cerebral arteries.
**Acute chest syndrome (ACS)**	A vaso-occlusive crisis of the pulmonary vasculature commonly seen in people with sickle cell anemia. This may include ACS incidence, ACS severity, ACS relapse rate, ACS duration.
**Neurocognitive function**	Cognitive functions associated with specific pathways or loci within the brain that are affected by different disease processes. Neurocognitive functions can be assessed by standard tests such as the Mini-Mental State Examination (MMSE) and the Montreal Cognitive Assessment (MoCA).
**Health-related quality of life (HRQOL)**	Patient's perception of the effect of their illness and its treatment on general well-being. HRQOL assessment for SCD should include measures of physical, social, and emotional functioning. Pain interference and fatigue should also be measured as part of HRQOL in SCD.
**Ability to return to usual activities**	Ability to carry out various activities such as going to work, doing housework, attending school, driving, shopping, etc.
**Frequency of hospitalization**	The number of admissions for a patient in a facility over a specified time period.
**ED/acute care visit**	The number of visits to the emergency department and/or SCD acute care center, where patients can receive timely treatment for urgent or extreme medical conditions.
**Need for blood transfusion**	This may include the number of transfusions, total units of transfusion, number of patients receiving transfusion.
**Cause-specific survival/mortality**	Impacts on survival/mortality caused by specific factors.
**Event-free survival**	The length of time after primary treatment for SCD ends that an individual remains alive and free of SCD symptoms.
